# Cancer driver drug interaction explorer

**DOI:** 10.1093/nar/gkac384

**Published:** 2022-05-17

**Authors:** Michael Hartung, Elisa Anastasi, Zeinab M Mamdouh, Cristian Nogales, Harald H H W Schmidt, Jan Baumbach, Olga Zolotareva, Markus List

**Affiliations:** Institute for Computational Systems Biology, University of Hamburg, 22607 Hamburg, Germany; School of Computing, Newcastle University, 2308 Newcastle upon Tyne, UK; Department of Pharmacology and Personalised Medicine, Maastricht University, 6229 Maastricht, Netherlands; Department of Pharmacology and Toxicology, Faculty of Pharmacy, Zagazig University, 44519 Zagazig, Egypt; Department of Pharmacology and Personalised Medicine, Maastricht University, 6229 Maastricht, Netherlands; Department of Pharmacology and Personalised Medicine, Maastricht University, 6229 Maastricht, Netherlands; Institute for Computational Systems Biology, University of Hamburg, 22607 Hamburg, Germany; Computational Biomedicine Lab, Department of Mathematics and Computer Science, University of Southern Denmark, 5230 Odense, Denmark; Institute for Computational Systems Biology, University of Hamburg, 22607 Hamburg, Germany; Chair of Experimental Bioinformatics, TUM School of Life Sciences, Technical University of Munich, 85354 Freising, Germany; Chair of Experimental Bioinformatics, TUM School of Life Sciences, Technical University of Munich, 85354 Freising, Germany

## Abstract

Cancer is a heterogeneous disease characterized by unregulated cell growth and promoted by mutations in cancer driver genes some of which encode suitable drug targets. Since the distinct set of cancer driver genes can vary between and within cancer types, evidence-based selection of drugs is crucial for targeted therapy following the precision medicine paradigm. However, many putative cancer driver genes can not be targeted directly, suggesting an indirect approach that considers alternative functionally related targets in the gene interaction network. Once potential drug targets have been identified, it is essential to consider all available drugs. Since tools that offer support for systematic discovery of drug repurposing candidates in oncology are lacking, we developed CADDIE, a web application integrating six human gene-gene and four drug-gene interaction databases, information regarding cancer driver genes, cancer-type specific mutation frequencies, gene expression information, genetically related diseases, and anticancer drugs. CADDIE offers access to various network algorithms for identifying drug targets and drug repurposing candidates. It guides users from the selection of seed genes to the identification of therapeutic targets or drug candidates, making network medicine algorithms accessible for clinical research. CADDIE is available at https://exbio.wzw.tum.de/caddie/ and programmatically via a python package at https://pypi.org/project/caddiepy/.

## Graphical Abstract

**Graphical Abstract:**
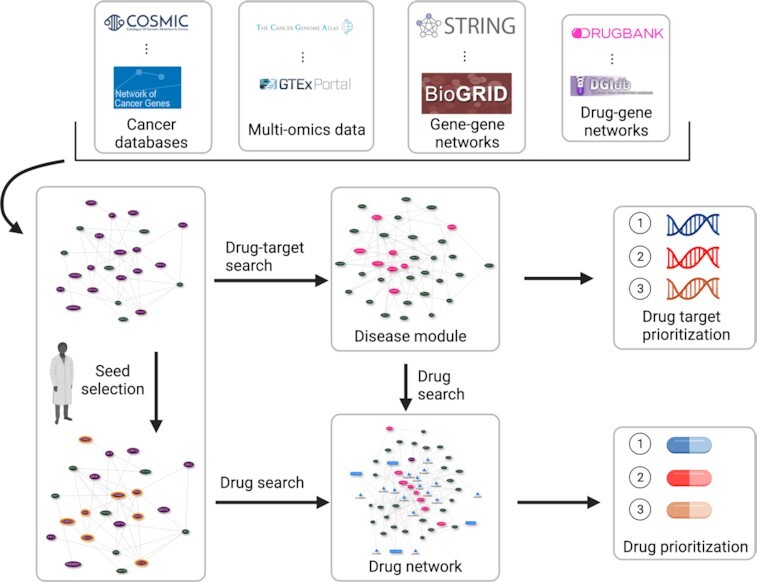
CADDIE: prioritize drug targets and identify drug repurposing candidates with network medicine algorithms in cancer.

## INTRODUCTION

Cancer is a set of diseases caused by alterations of the genome that lead to unregulated cell growth. Cancer therapy development is challenged by the heterogeneity of the disease, where similar phenotypes show distinct somatic mutations (1). Conversely, different cancer entities often have shared molecular mechanisms that can be exploited in network pharmacology (2). Since anti-cancer drugs cause extensive side effects, it is important to target molecular lesions and affected pathways specific to a tumor (3,4) and to adapt treatment in response to drug resistance (1). To identify suitable drugs, classical drug development is unsuitable as it is time consuming, expensive and often terminates in failure (5). A fast and cost-effective alternative to drug development is drug repurposing, the search of potentially effective drugs among the drugs approved for other indications (3).

Drug repurposing candidates can be identified computationally, experimentally or synergistically, where computational approaches lead to a hypothesis which can be validated experimentally (6). Network-based algorithms have proven successful for drug repurposing and to detect possible off-target effects (7). Computational drug repurposing methods based on protein-protein interaction (PPI) networks have been applied to find possible drugs against SARS-CoV-2 (8,9). In cancer, network-based repurposing allows to predict and prioritize drugs particularly for a group of cancer driver genes (10). However, some driver genes like MYC in ovarian cancer (11) are not druggable directly, suggesting the application of network-based approaches for identifying druggable interaction partners in the disease module, a local neighborhood of genes linked to the disease mechanism. Moreover, it has been shown that network-based drug repurposing makes predictions that align with the outcome of current clinical drug studies and enables accelerated drug development in cancer (12).

Network-based drug repurposing approaches have proven successful for individual cases but in oncology, these are only accessible to researchers with advanced programming skills (13,14). To make these methods available to a broader scientific community, we present the Cancer Driver Drug Interaction Explorer (CADDIE). CADDIE generates hypotheses for drug development and repurposing in cancer with a graphical user interface that thus not only bioinformaticians or researchers with a computer science background can use this platform but also clinicians and biologists who are looking for candidates for clinical trials or cell line studies. It offers comprehensive gene and protein information collected from different resources as well as an interactive network visualization to inform the selection of seed genes or proteins, i.e. putative drug targets that have been implicated in a cancer type or in an individual via genetic screening and serve as a starting point for network-based drug target drug repurposing candidate discovery. The human interactome can be queried using a number of algorithms and user-defined targets, such that each individual case can be investigated. Users can enter a list of seed genes or proteins and choose among seven different algorithms to prioritize 16761 genes and 6840 drugs.

### Comparison with existing tools

A number of in silico tools have been developed for drug repurposing. ACID (15) creates predictions based on individual 3D structures of molecules but can not work with a list of genes or proteins, does not consider second-order interactions in a network approach, or visualize the interactions to enhance the interpretability of the outcome. Gene2Drug (16) predicts gene-drug interactions based on pathway information, but fails to produce results for typical oncogenes such as ‘KRAS’, ‘PTEN’ or ‘TP53’. Further, it is not possible to search drugs for multiple query genes, and similar to ACID, indirect interactions are not taken into account. NeDRex (10) is a disease-agnostic network medicine platform for disease module identification and drug repurposing. CoVex (9) provides similar functionalities as CADDIE but is limited to SARS-CoV-2. While none of these tools is tailored towards research in oncology, CADDIE applies its network medicine algorithms to ten human gene-gene and drug-gene interaction databases with annotated cancer driver genes, cancer-type specific mutation frequencies, gene expression information, genetically related diseases, and approved anticancer drugs. Thus, in comparison to NeDRex and CoVex, CADDIE allows the user to select the datasets of interest for transparency, reproducibility and comparability. Also, usage of drugs in cancer and related clinical trials is highlighted by integrated datasets, facilitating interpretation of the results in the cancer context The comprehensive visualization in CADDIE allows for intuitive explorative analysis and highlights the interplay between interacting molecules. The network algorithms traverse the complete human interactome searching for directly as well as indirectly operating drugs with the option to consider integrated omics data as edge weights.

## MATERIALS AND METHODS

### Database

The CADDIE database integrates six gene–gene interaction (GGI) and four drug-gene interaction (DGI) datasets (see Supplementary Material for details). Each dataset provides interactions collected from different sources, e.g. peer-review, text-mining or based on *in silico* predictions (see Table 1).

**Table 1.** Integrated interaction databases and their incorporated edge types

Name	Data type	Experimental	Literature	Predicted
NCG6	CDG	+	+	+
COSMIC	CDG	+	+	-
IntOGen	CDG	-	-	+ (pipeline)
cancer-genes.org	CDG	-	-	+ (MutPanning)
BioGRID	GGI, DGI	+	+	+
STRING	GGI	+	+	+
APID	GGI	+	-	-
IID	GGI	+	-	+
HTRIdb	GGI	+	-	-
Reactome	DGI	-	+	-
DrugBank	DGI	-	+	-
ChEMBL	DGI	+	+	-
DGIdb	DGI	+	+	-

List of databases implemented in CADDIE. Data types are cancer driver gene (CDG), gene-gene interaction (GGI), and drug-gene interaction (DGI). GGIs encompass interactions of corresponding proteins.

CADDIE integrates the four cancer driver databases COSMIC (17), NCG6 (18), IntOGen (19) and cancer-gene.org (20) (see Supplementary Figure S1) and mutation frequency data from TCGA to assist users in seed selection. Information on drugs that are classified as ‘antineoplastic or immunomodulating’ according to the Anatomical Therapeutic Chemical (ATC) classification system was downloaded from the WHO (https://www.whocc.no/atc_ddd_index/) in November 2020. We further created a curated list of approved cancer drugs (CanceRx), which was extracted from the National Cancer Institute website (https://www.cancer.gov/about-cancer/treatment/drugs) and Cancer Research UK website (https://www.cancerresearchuk.org/about-cancer/cancer-in-general/treatment/cancer-drugs/drugs) between 17–26 January 2022. Drugs lacking a direct known protein target such as antimetabolites, alkylating agents together with palliative drugs, e.g., those used to treat anemia or chemotherapy induced nausea and vomiting, were not considered. This allows users to distinguish drugs already used as standard of care (SOC) therapy from newly suggested repurposable drugs. Drugs from the Cancer Therapeutics Response Portal (https://portals.broadinstitute.org/ctrp/, version 2) are also implemented in the CADDIE database (21). The drug search can be limited to these drugs, such that all returned drugs were evaluated in the context of cell line studies.

### Seed selection

Seed genes are the starting nodes for all implemented network algorithms and thus crucial for the quality of the results. Users can construct a set of seed genes for a selected cancer type based on integrated mutation and tissue-specific gene expression information (see Supplementary Figure S4). Genes with more frequent cancer mutations and high expression are prime drug target candidates (22). Seed genes also can directly be extracted from variant calling files using PolyPhen-2 (23). Lastly, seeds can be selected based on related diseases or uploaded as a list.

### Algorithms

**Figure 1.**
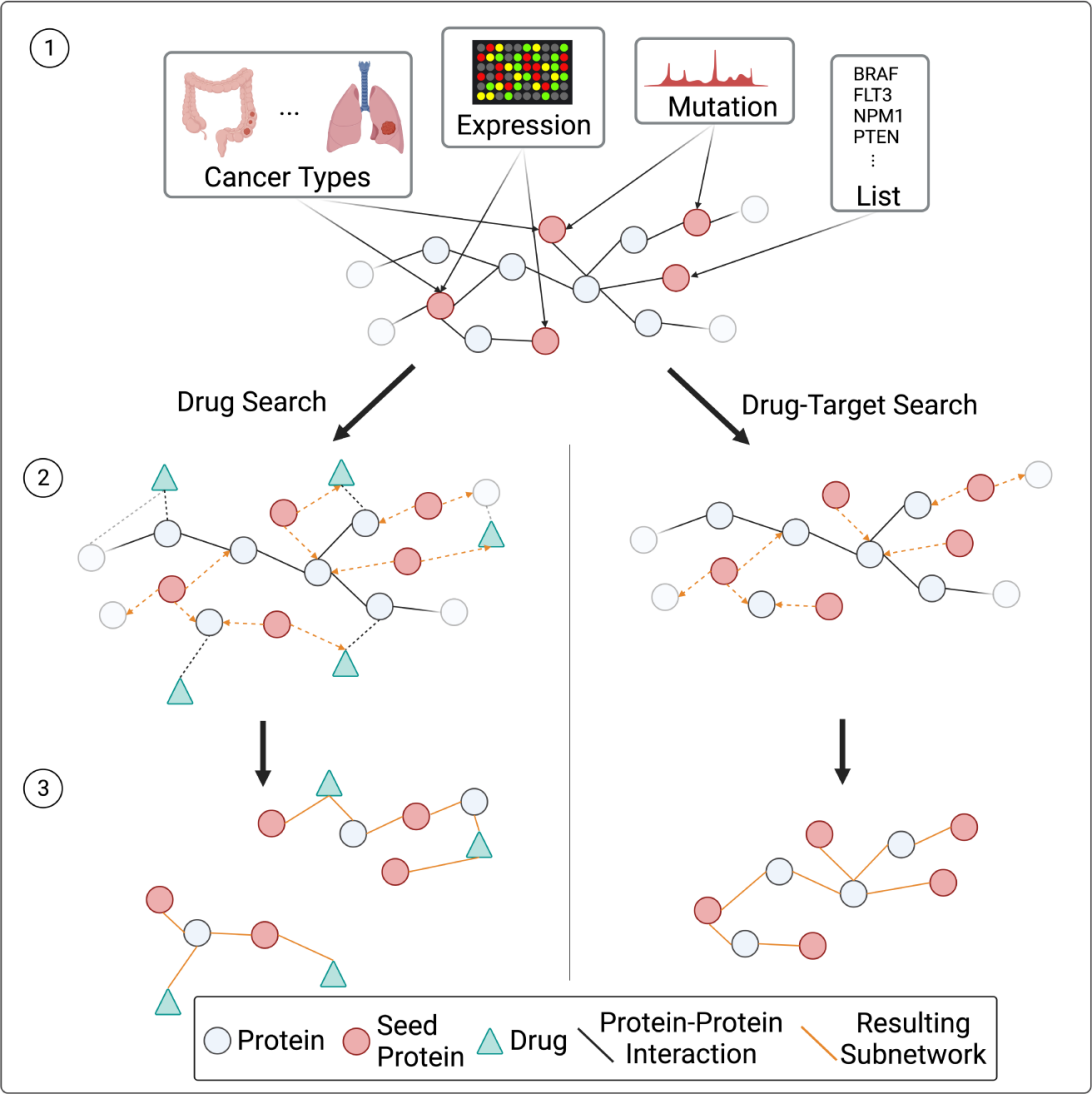
Seed genes for drug target and drug search can be selected from integrated cancer driver databases, via mutation or expression data from TCGA and GTEx using user-defined thresholds, or by uploading a list of genes (1). Based on the seed genes, putative drugs or drug target candidates are identified using a broad selection of network medicine algorithms that traverse the human gene-drug interactome (2). While the drug search reports drugs in proximity to the seed genes, the drug target search returns genes interacting with the disease module spanned by the seed genes (3).

CADDIE prioritizes drugs and targets based on their network properties and integrates seven network-based algorithms for both drug target identification and drug repurposing/prioritization (see Table 2) which were previously suggested for drug repurposing in SARS-CoV2 (9). Six different algorithms identify putative drug targets most central to the seed genes in the GGI network and thus indicative of functional relationships (see Figure 1). Four algorithms determine drug candidates in proximity to the seed genes in the DGI network, hence drugs that may have indirect influence on the seed genes. As many putative cancer driver genes are difficult to be targeted directly (11), users may consider running a drug target search prior to a drug search.

**Table 2.** Algorithms for drug target identification and repurposing

Name	Drug target prioritization	Drug repurposing
TrustRank	X	X
Degree Centrality	X	X
Harmonic Centrality	X	X
Betweenness Centrality	X	
KeyPathwayMiner	X	
Multi-level Steiner Tree	X	
Network Proximity		X

Listed are the seven integrated network algorithms used for drug target prioritization and drug repurposing with their respective application cases.

Multi-level Steiner Tree (24), KeyPathwayMiner (25) and betweenness centrality can be considered as aggregation approaches as they connect the seeds by finding nodes in between them. The other algorithms are propagation methods which traverse deeper into the network starting from the seed genes. Only the propagating algorithms can identify drug candidates as the drugs lie in the network outside of the disease module. In comparison to the other drug search algorithms, Network Proximity (26) is the most explorative algorithm by design, propagating deeper into the network to propose drugs that are linked to the neighborhood of the seeds.

Due to efficient preprocessing of the networks with graph-tool (https://graph-tool.skewed.de/, version 2.32), the complete human gene-drug interactome can be searched for the optimal results in real-time. The graph-tool module makes it possible to benefit from the speed advantages of C++ while still having the simplicity of python code.

The GGI and DGI networks were augmented with the expression and mutation data stored in the CADDIE database. Using this data as edge weights, the network algorithms can account for the differences in gene expression and somatic mutation frequencies across tumor types. CADDIE further allows users to restrict DGIs to activators or inhibitors. The choice of algorithm and its parameters remains up to the user. It is often beneficial to run multiple analysis tasks with varying settings to cover a broader range of solutions. For this purpose, a task summary function combines the results into one single network while node counts give an intuitive understanding of the importance.

### Programmatic access

The CADDIE backend with a task server and the database offers an application programming interface (API) to exchange data with other services. A python package was created to provide a programmatic interface to CADDIE’s drug repurposing functionalities. It allows running a large number of tasks using different algorithms and parameters in custom workflows for sophisticated analyses as part of other tools. CADDIE is further available as docker container which encapsulates all dependencies and can be deployed locally to overcome privacy concerns w.r.t. sensitive genomic data (27).

## RESULTS

### CADDIE web interface

CADDIE is an open access online platform for prioritizing (repurposing) putative drug candidates or targets in cancer using network medicine methods on the combined human gene-gene and drug-gene interactome. Results are visualized online and can be downloaded for further processing. The web interface is freely available and no login is required.

The main feature is the Explorer (see Supplementary Figure S2), which allows users to visualise the interactome of genes and consider known cancer driver genes to find seeds for drug target identification and drug prioritization. Analyses can be triggered with default values or with customized parameters for fine-tuning. Since the full GGI network with 16761 genes would clutter the view, CADDIE shows only cancer type-related genes connected through a minimum spanning tree (see Supplementary Figure S3) (28).

The GGI network visualizes the interactions of the cancer driver genes, helping the user to define a selection of seed nodes that will then be considered for the drug target or drug search. CADDIE also displays complementary information about mutation rates, gene expression, comorbidities, and related cancer types (see Supplementary Figure S4).

Users can choose among several network medicine algorithms (see Supplemental Material for details) for identifying direct or proximal drug targets and related drug (repurposing) candidates. The analysis view shows the results, i.e. genes ranked by their importance according to a user-selected network centrality measure. Detailed information about drugs is highlighted. CADDIE also contrasts importance scores with the node degrees in the interactome (see Supplementary Figure S5 and Supplementary Table S1). This is due to the fact that nodes with a high degree are more likely to be selected by chance, whereas nodes with a low degree and a high score could be more specific to the disease and thus represent attractive targets. All used parameters are displayed for reproducibility. Task results can be shared by copying the URL and all data can be downloaded.

### Showcase sarcoma

**Figure 2.**
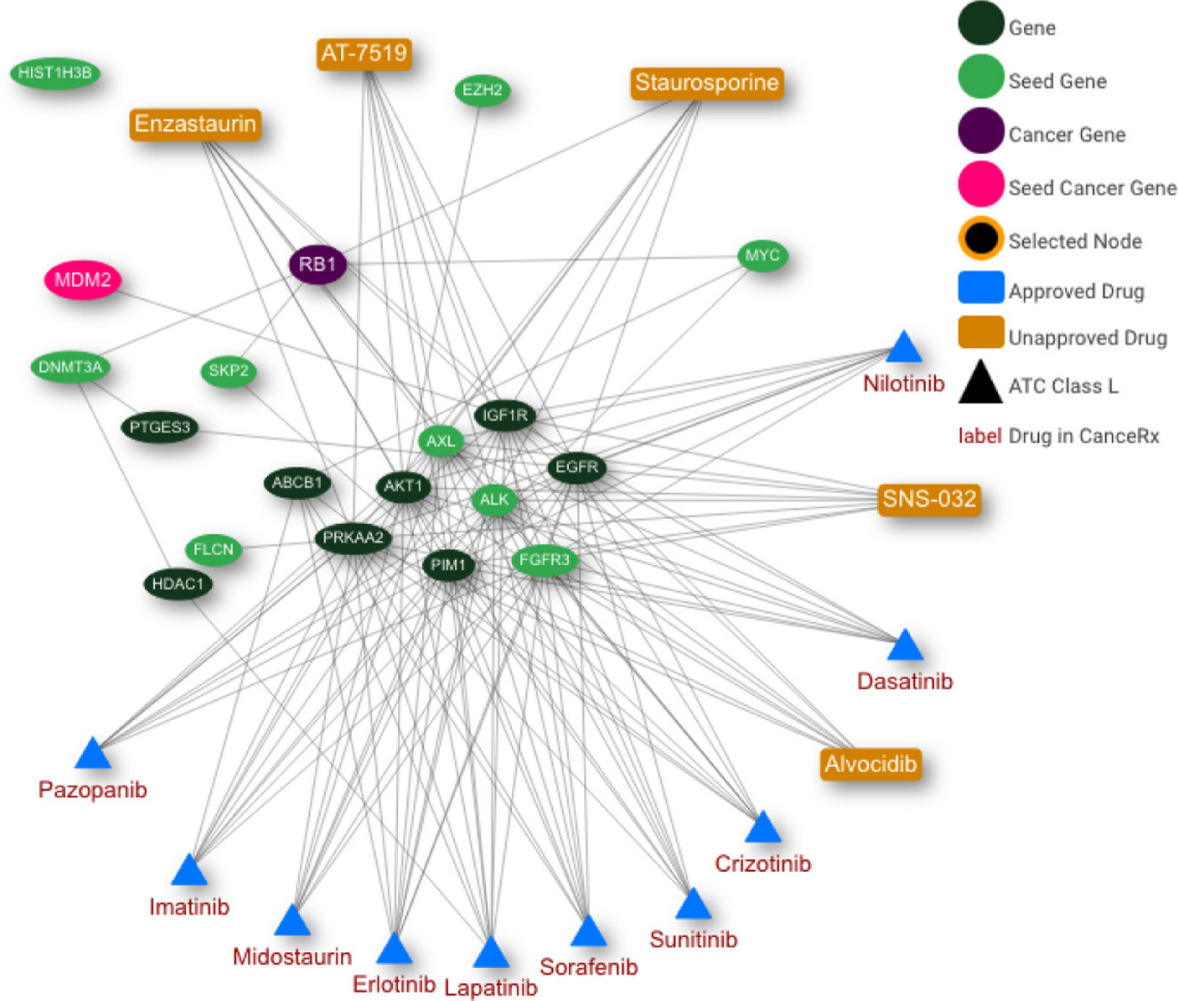
Drug network computed with TrustRank for sarcoma with BioGRID gene–gene and drug–gene interactions. The cancer driver gene information is taken from COSMIC.

Ohshima *et al.* identified cancer driver genes in 16 cancer types based on their frequent amplification and overexpression (29). Their study highlighted sarcoma where AXL was reported as drug target. Based on this finding, we selected the identified genes DNMT3A, FGFR3, ALK, EZH2, HIST1H3B, MDM2, SKP2, MYC, AXL and FLCN as seed genes in CADDIE. We further chose BioGRID (30) as GGI and DGI dataset and selected the sarcoma cancer type from COSMIC to highlight known drivers for this cancer type. Next, we used the TrustRank algorithm (limited to the top 15 drugs with the highest scores, default parameters otherwise) where we included indirect and unapproved drugs for an exhaustive result (see Figure 2).

In the resulting network, we notice that the seed genes AXL, ALK and FGFR3 are grouped in the center of the graph as they have interactions with all of the reported drugs. RB1, a gene classified as a driver in sarcoma by COSMIC (31), is also well connected in our solution, linking protein kinase C inhibitor staurosporine (32) to the seed genes MYC, SKP2 and DNMT3A.

The highest scored drugs imatinib (see Table 3) as well as sorafenib, sunitinib, dasatinib, nilotinib, pazopanib and crizotinib have been studied in sarcoma and are partially approved by the FDA for this use case (33). Further, CADDIE assigned the second highest score to staurosporine, an investigational drug with apoptosis-inducing abilities and synthetic analogs as anticancer drugs in clinical trials (32,34), suggesting it as a putative drug for further research.

**Table 3.** Drug search results for sarcoma

Name	Approved	ATC L	CanceRx	Score	Degree
Imatinib	yes	yes	yes	1	413
Staurosporine	no	no	no	0.995	393
Sorafenib	yes	yes	yes	0.991	401
Lapatinib	yes	yes	yes	0.984	387
Sunitinib	yes	yes	yes	0.983	388
Alvocidib	no	no	no	0.98	381
Dasatinib	yes	yes	yes	0.978	381
Nilotinib	yes	yes	yes	0.978	386
AT-7519	no	no	no	0.977	377
SNS-032	no	no	no	0.976	376
Erlotinib	yes	yes	yes	0.976	381
Pazopanib	yes	yes	yes	0.975	375
Crizotinib	yes	yes	yes	0.974	377
Midostaurin	yes	yes	yes	0.974	377
Enzastaurin	no	no	no	0.974	376

Listed are the top 15 drug results reported by CADDIE for sarcoma. Each row contains the drug name, approval by FDA, EMA or HC, whether it is listed as antineoplastic or immunomodulating agent (ATC class L) by the WHO, whether it is contained in CanceRx, the normalized score and the node degree in the drug–gene-interactome (BioGRID).

CDK-9 is a previously reported drug target in sarcoma (35). The CDK-9 inhibitors alvocidib and SNS-032 have shown effective in treating Ewing sarcoma cells (36,37). Interestingly, the drug AT-7519, also a CDK-9 inhibitor, has not been considered in sarcoma while it is already undergoing clinical trials as an anticancer drug in different cancer types (38).

The approved drug lapatinib has shown modest effectiveness in a phase 2 study in a sarcoma subtype (39). Another approved drug is erlotinib, which has shown significant growth delay in a sarcoma xenograft model (40). Approved drugs represent valuable repurposing candidates as their safety is already tested.

## CONCLUSION

For the future, we plan to extend CADDIE with additional network analysis methods (41) and data sources, e.g. oncology data such as cell line study information from PharmacoDB (42) to improve interpretability to the meaning of the predictions. Antineoplastic agents have a broad spectrum of targets and the cause of the intended effect often remains unclear. Databases reporting on cancer driver genes label cancer types differently and show differences in granularity for cancer subtypes. While this lack of harmonization poses a challenge for the users of CADDIE, we decided against limiting the search of seed genes to broadly accepted labels. In summary, CADDIE is the first drug repurposing platform tailored towards oncology. It offers access to a broad set of resources and allows biomedical researchers to suggest and prioritize drug targets and to identify suitable drug repurposing candidates using state-of-the-art network medicine algorithms.

## DATA AVAILABILITY

The authors declare that all data supporting the findings of this study are available publicly and their integration is described accordingly within the paper and its supplementary information file. The CADDIE code is available on GitHub (https://github.com/biomedbigdata) in the repositories CADDIE-frontend, CADDIE-backend and caddiepy.

## Supplementary Material

gkac384_Supplemental_File
