# Supplementary material for: Cancer driver drug interaction explorer
Source: Nucleic Acids Res. 2022 May 17;50(W1):W138–44. doi: 10.1093/nar/gkac384 (PMC9252786; doi:10.1093/nar/gkac384)
Supplement: gkac384_Supplemental_File [file gkac384_supplemental_file.pdf]

Supplementary Material for CADDIE

1 Implementation

A PostgreSQL database (version 12.2) was created with cancer-related data, therefore the API, built with the Django web framework (version 3.0.5) with Python (version 3.6) and the Django REST framework (version 3.11.0), was changed respectively. The algorithms were implemented in graph-tool (version 2.3.1) (1).

The layout in the frontend has been implemented using Angular (version 9.0.2). The genetically related diseases are displayed as a bar chart using the JavaScript library Plotly (version 1.56). Further, the network has been implemented with the JavaScript libraries vis-data (version 6.5.1) and vis-network (version 7.4.2).

2 Databases

The implemented selection includes interactions supported by different levels of evidence, from experimentally validated to predicted interactions. In total, four resources for cancer driver genes, six for GGIs, and four for drug-gene interactions (DGIs) were integrated (see Table 1). Genes and proteins as well as GGIs and PPIs are treated as equivalent.

In this section, first, the cancer driver databases are described, then the GGI followed by the DGI databases. In the end, other resources for diseases and mutations are mentioned.

2.1 NCG6

The Network of Cancer Genes (NCG6, http://ncg.kcl.ac.uk/, version 6) maintains a manually curated collection of predicted cancer driver genes based on somatic mutations and literature (2). The annotations of cancer types for 2088 cancer driver genes were integrated.

2.2 COSMIC

The Catalogue Of Somatic Mutations In Cancer (COSMIC, https://cancer.sanger.ac.uk/cosmic, version 92) is the largest database for somatic cell mutations in human cancer (3), containing manually curated data supplemented with data from other major cancer data portals and literature. 723 cancer driver genes and their related cancer types were extracted from the Cancer Gene Census.

2.3 IntOGen

The Integrative OncoGenomics (IntOGen, https://www.intogen.org/search, version 2020-01-01) framework identifies cancer driver genes based on somatic mutations in sequenced tumor samples (4). 962 cancer driver genes and their corresponding cancer types were included.

2.4 Cancer-genes.org

Cancer-genes.org (http://cancer-genes.org/) incorporates the statistical framework Mutpanning (5) which aims to discover novel cancer driver genes based on frequencies and nucleotide context of somatic mutations. 456 cancer driver genes and their cancer types were added from this resource.

2.5 BioGRID

The Biological General Repository for Interaction Datasets (BioGRID, https://orcs.thebiogrid.org/, version 4.0.189) is a public biomedical database, containing genomes, proteomes and interactomes of a wide range of organisms. Its data is obtained by text-mining approaches and direct user submissions, where all interactions are based on experimental evidence (6). BioGRID provided 319747 gene-gene interactions and 52548 drug-gene interactions in human.

2.6 STRING

STRING (http://string.embl.de/, version 11.0) is a repository of direct and indirect protein-protein interactions, including predicted interactions and interactions with experimental evidence (7). All 211712 interactions in human with experimental evidence were taken from STRING.

2.7 APID

The Agile Protein Interactomes DataServer (APID, http://cicblade.dep.usal.es:8080/APID/init.action, version January 2019) aims for experimentally validated protein-protein interactions by data mining from literature, selected databases, as well as 3D structure predictions (8). All 130578 interactions of the human interactome with at least two experimental pieces of evidence were integrated from this resource.

2.8 IID

The Integrated Interactions Database (IID, http://iid.ophid.utoronto.ca/, version 2018-11) offers context-specific protein-protein interaction networks experimental evidence and high-confidence predictions (9). The 224256 cancer-related PPIs related to human were extracted from IID.

2.9 HTRIdb

The Human Transcriptional Regulation Interactions database (HTRIdb, http://www.lbbc.ibb.unesp.br/htri/) targets transcriptional regulation interactions based on experimental validation (10). 38761 protein-protein and transcription factor-target gene interactions were taken from HTRIdb.

2.10 Reactome

Reactome (https://Reactome.org, version 74) is a pathway knowledgebase that grows via a manual curation and peer review process (11). The complete human interactome with 17676 PPIs was extracted from Reactome.

2.11 DrugBank

DrugBank (www.drugbank.ca, version 5.1.7) contains interaction data about all drugs approved by the United States Food and Drug Administration (FDA), European Medicines Agency (EMA) and Health Canada (HC) as well as drugs that are in the approval process (12). Information about 15445 drug-gene interactions as well as data about drug classes was integrated from DrugBank.

2.12 ChEMBL

The ChEMBL database (https://www.ebi.ac.uk/chembl/, version 27) contains knowledge about bioactive drug-like small molecules and describes their interplay with gene and protein targets (13). 53012 drug-gene interactions as well as ID mapping tables were used from ChEMBL.

2.13 DGIdb

The Drug Gene Interaction Database (DGIdb, https://www.dgidb.org, version 3.0) collects knowledge about the druggability of the genome from literature and web resources (14). Its 26201 drug-gene relations were added as an additional source.

2.14 NeDRex

NeDRex (https://nedrex.net/, version 1.45.0) is a multi-omics data resource for drug repurposing and disease module identification (15). It was used to fetch 8237 diseases related to all genes implemented in CADDIE as well as drug information. The drugs include a status information which indicates the approval status in FDA, EMA or HC.

2.15 Expression and Mutation Data

The Genomic Data Commons (GDC, https://gdc.cancer.gov/, November 2020) is part of the National Cancer Institute (NCI) and is dedicated to building a cancer genomic database (16). Cancer type-specific expression and somatic mutations data from The Cancer Genome Atlas (TCGA) (17) was taken from this source and a mutation count mapping was generated for each gene. A mutation score was calculated by counting the different mutations for each gene and applying a logarithmic normalization. Further, Tissue specific expression data was obtained from GTEx in November 2020 (18).

2.16 Comparison of integrated datasets

Numerous data resources are available for each cancer driver genes, gene-gene interactions, and drug-gene interactions, allowing to apply the methods on personalized input data (see Table 1). Since many methods have been proposed to predict cancer driver genes, without the existence of an acknowledged gold standard, each one of them has its legitimation and drawbacks (19), leading to many predictions represented by different databases. Upon taking a closer look at the data, the differences show. From the used cancer driver gene datasets, NCG6 with 2088 driver genes is the largest one, while the other datasets have all less than 1000 driver genes. Only 182 cancer driver genes occur in all datasets while 1390 occur only in NCG6 and not in any other implemented cancer driver dataset, displaying the discrepancy in the methodological approaches (20) with which these resources were created (see Figure S1).

BioGRID, even though it provides a high number of interactions for both GGIs and DGIs, tends to have comparably little unique interactions. This is because BioGRID incorporates many other databases. The peer-reviewed GGI data resources Reactome and HTRIdb with solely experimentally validated interactions have with 17676 and 38761 interactions, respectively, the least interactions. Therefore, the variety of data resources in CADDIE offer the freedom of selecting the input data of choice between different data standards.

3 Algorithms

A number of different graph-based algorithms are implemented in CADDIE. They reach from simple centrality measurements to more complex approaches like TrustRank (Gyöngyi et al., 2004) and Network Proximity (21), where each of them can again be modified by the user via a set of algorithm-specific input parameters. While the algorithms all differ in their methodology to allow for a variety of comparable results and use case-specific solutions, they commonly expect user-selected seed nodes. Overall four algorithms for the drug search and six for the drug target search are available.

3.1 Degree Centrality

Degree centrality is a measure used for ranking the nodes in a network. It is defined as the number of neighbors a node has divided by the number of nodes (22). It is implemented in CADDIE as

$$centrality_{degree}(x) = deg(x)$$

where x is a given node and $deg(x)$ is its degree. While it is a commonly used network analysis technique (23), it most importantly has been shown useful in the identification of essential proteins in PPI networks (24). Thus, it is a simple approach to classifying the network-related importance of a particular protein. In CADDIE it can be used to discover valuable drug targets or drugs, based on the seed selection given by the user.

3.2 Harmonic Centrality

Harmonic centrality can be measured as the average shortest distance from each node to all other nodes in a network. This measurement is the equivalent of closeness centrality (25) for disconnected graphs (22, 26). Formally speaking, it can be annotated as

$$centrality_{harmonic}(x) = \sum_{y \neq x}^{y} \frac{1}{dist(x, y)}$$

where x is a given node and $1/dist(x, y) = 0$ if $dist(x, y) = \infty$ (22). The closer a node is to other nodes, the higher the score. It has already been proven successful in a number of biological network problems for instance with metabolic (27) or PPI networks (28). In CADDIE, this algorithm can be used to find drug targets or drugs that affect the seed nodes as direct as possible.

3.3 Betweenness Centrality

Betweenness is obtained by finding the shortest paths for each pair of nodes in the network and assessing the number of shortest paths that pass through a particular node, such that a measure of the centrality of a node in a network global context is received (27, 29). Betweenness Centrality has been established as a common measurement in network biological application (30) and is especially practical in finding communities in large networks (31). In

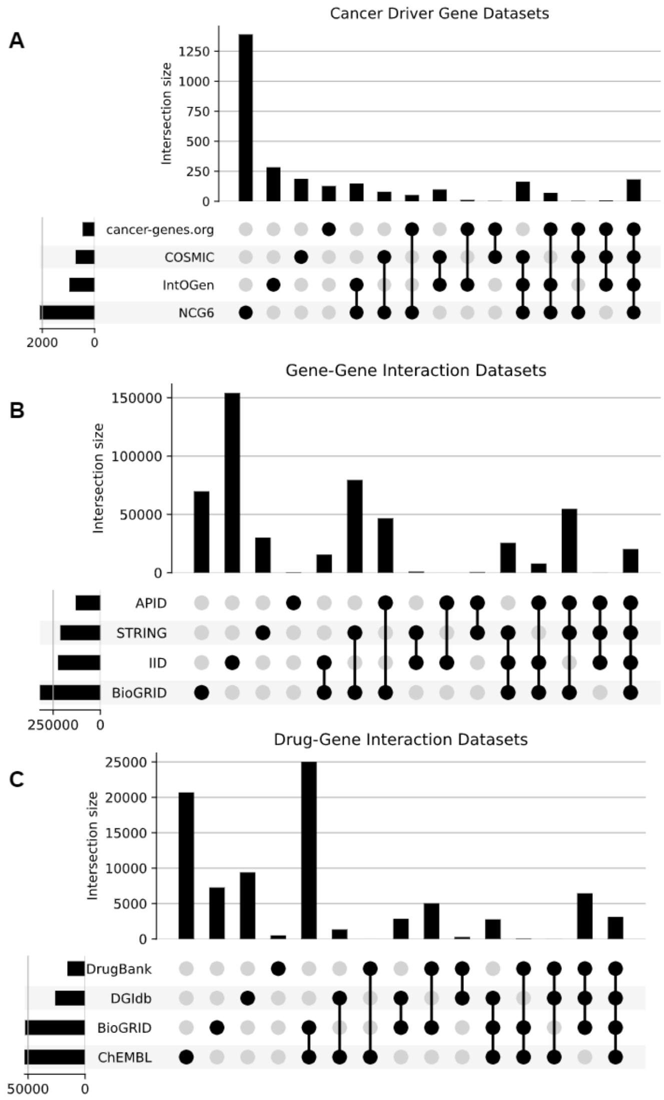

Figure S1: Comparison of datasets
This figure describes the intersections and uniqueness of the datasets available in CADDIE in each topic (cancer driver genes (A), gene-gene interactions (B), and drug-gene interactions (C)). Reactome and HTRIdb were left out for the gene-gene interactions due to the very low number of interactions.

CADDIE, betweenness is based on the shortest paths between the seed nodes only and can be used to find drug targets with maximized connectivity to all seeds (32).

3.4 TrustRank

TrustRank is based on the same concepts as the Google PageRank algorithm (Page et al., 1999) and Closeness Centrality. A crawler searches the network based on user-selected seeds and ranks visited nodes, damping the score based on the distance traveled. The damping factor can be set by the user in a range from 0-1, with a higher damping factor causing the crawler to explore nodes in close proximity or in larger portions of the GGI network. In CADDIE, TrustRank is able to find putative drug targets as well as drug candidates.

3.5 Network Proximity

Network Proximity, as introduced by Guney et al. (21), is the average length of shortest paths from drugs to all neighboring nodes to the user-selected seed nodes. The algorithm then computes a statistical significance score comparing against random expectation. This algorithm was adopted in CADDIE so that best-scored drugs are returned to the user as candidate drugs.

3.6 KeyPathwayMiner

Network enrichment analysis is the search of subnetworks of GGI networks based on common biological functions (33). KeyPathwayMiner (KPM, https://keypathwayminer.compbio.sdu.dk/keypathwayminer/, version 5) is an online tool developed by Alcaraz et al. (34) for pathway enrichment analysis. Users have the option to utilize KPM for their drug-target search by selecting seed nodes in the GGI network and letting KPM find an interaction network of genes that are commonly dysregulated spanned by the seed genes. The resulting proteins are presumably functionally related to the seed nodes, and therefore are suitable drug-target candidates. Only one parameter K has to be set by the user which defines the amount of permitted intermediate nodes that are neither part of the seed nodes nor the common pathway.

3.7 Multi-level Steiner Tree

Computation of a minimum spanning tree is a very well known combinatorial problem, which is defined as finding a network that connects all the nodes and minimizes the global length (35). Since the calculation of a minimum spanning tree is an NP-hard problem, the Multi-level Steiner Tree algorithm is used here to approximate the solution and solve the problem in reasonable time (36). It can be used to create a minimum spanning subnetwork between user-selected seed nodes, which happen to be central interaction partners between the seed nodes, and thus represent favorable drug-targets.

3.8 Summary

The summary function allows to combine different task results. It merges the individual resulting networks and creates statistics to conclude the different searches.

4 Explorer

The Explorer is the main part of the application. It allows the user to explore the interactome of your cancer type or cancer driver genes of interest. Most importantly, it provides a variety of information like related diseases or expression values to help selecting seed genes for drug or drug-target search.

4.1 Left Menu

On the left side, the user can find a menu bar that allows to select cancer types or gene interaction resources of interest. It also provides a short overview of your cancer type of interest. After selecting seed genes, the left menu allows the user to start analysis tasks (max. 3 at a time). The tasks will be displayed at the bottom of this menu in the "Tasks" section. A click on a finished task leads to the analysis view.

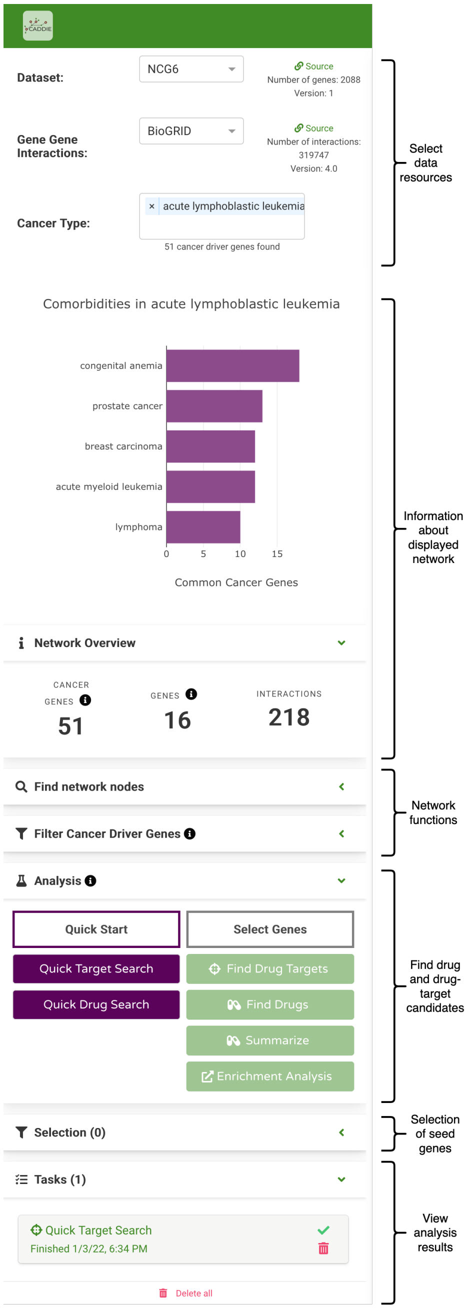

Figure S2: CADDIE left side menu.
The menu contains panels with different functionality. From here, the network can be edited and drug target search and drug prioritization algorithms can be started.

4.2 Gene-Interaction Network

On the right side of the explorer is the gene-interaction network. It contains all cancer driver genes of the selected cancer type(s), forming a minimal spanning tree in the human gene interactome. It is supplemented by genes found by the Multiple Steiner Tree algorithm, which was used to pre-compute the network (see Figure S3). Therefore, the network will change when a new interaction dataset is selected. The network gives an overview of the relations of the cancer driver genes to each other and lets the user select seed genes for the drug target or drug search by double-clicking the nodes. A single click on a node opens a window at the top of the left menu with detailed information about the drug or gene such as related diseases or the node degree in the complete human drug-gene interaction network.

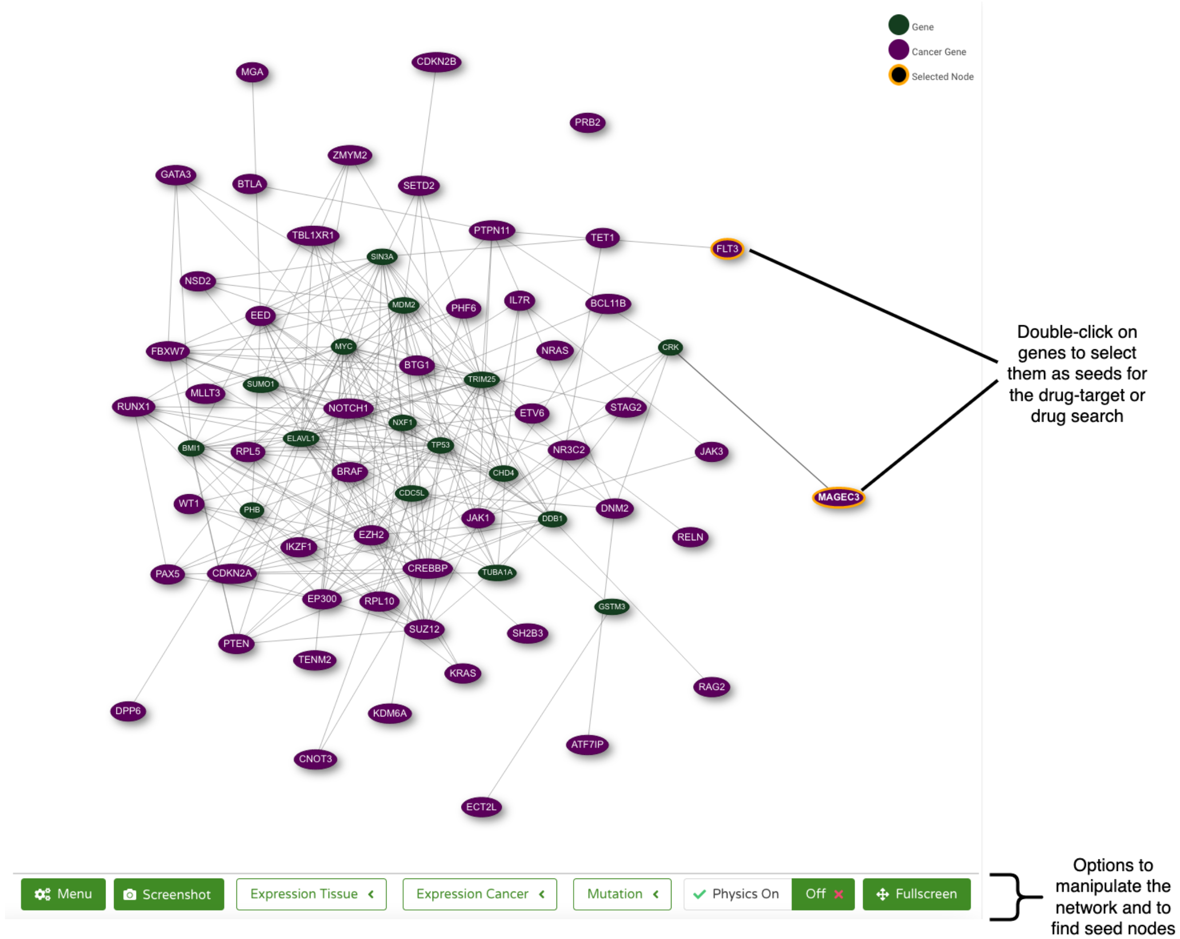

Figure S3: Interactive gene-gene interaction network.
Visualized is the gene-gene interaction network for all acute lymphoblastic leukemia driver genes stored in the NCG6 database. The Multiple Steiner Tree algorithm was applied together with the BioGRID interactions to find the edges and intermediate nodes.

Below the network, the user can find various functions that help to manipulate the network and to find genes of your interest. The expression of genes in different tissues (data from GTEx) as well as in different cancer types (data from TCGA) can be highlighted and genes may be selected automatically above a certain threshold. By e.g. selecting a cancer type here, the expression values will be loaded into the network and visualized via a color gradient. Now, via the function "Cancer expression" in the left menu or the menu below the network, genes can be added to the selection by setting an expression threshold (see Figure S4). The same possibility exists for mutation data from GDC. Furthermore, a menu is available with versatile options such as selection of all cancer driver genes or upload of a vcf-file to filter for genes with damaging mutations. The user can add more genes to the network by uploading a list seed genes (for example via the 'Custom genes' function in the left menu) followed by the 'Add to network' functionality. Gene-gene interactions are taken from the selected interaction database.

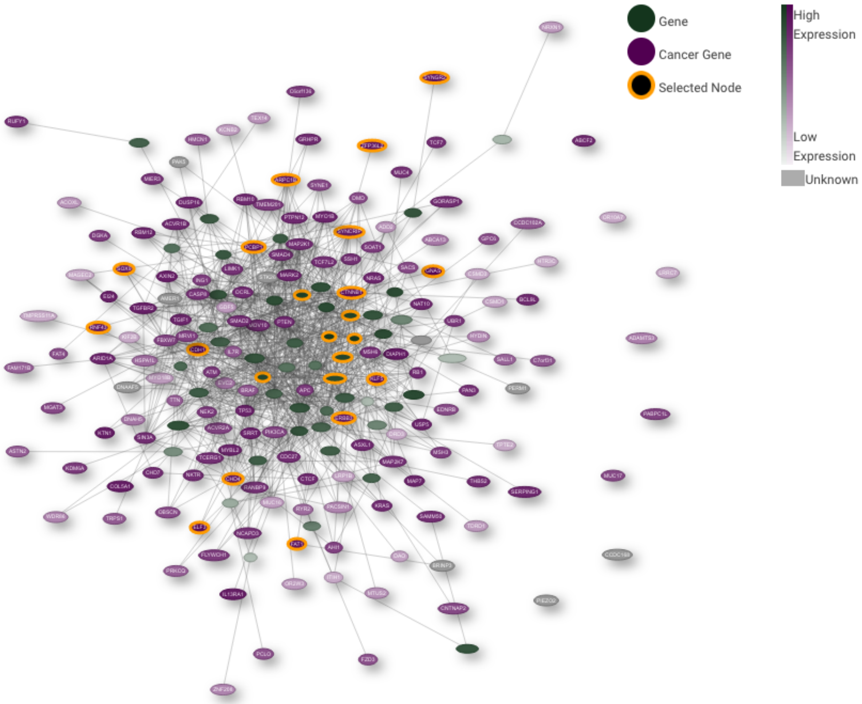

Figure S4: Colon adenocarcinoma expression visualized in the BioGRID gene-gene interaction network for the colorectal adenocarcinoma driver genes from NCG6. The cancer type selected to highlight expression was colon cancer. The expression threshold less than 12 transcript per million (TPM) selects 22 seed genes.

5 Showcase Ovarian Cancer

We apply CADDIE to ovarian cancer by selecting PPM1D, BARD1, CASP3, LRP1B, and CSMD3 - five of the most frequent drivers in ovarian cancer according to COSMIC dataset (3) - as seed genes. For a more conclusive drug candidate search, the KeyPathwayMiner drug target search (parameter K=5) was applied. The genes BRCA1, nucleoporin 43 (NUP43), MYC, and ubiquitin C (UBC) were returned. BRCA1 is a gene known to be associated with ovarian cancer (37), as is MYC (38), although MYC has shown to be difficult to target directly by drugs. Therefore, Zeng et al. have proposed CDK7, CDK12, and CDK13 as targets to down-regulate MYC upon inhibition. Targeting UBC in tumor cells with inhibited ubiquitin B has led to a significantly prolonged survival time (39) and members of the nucleoporin family have been associated with the susceptibility of chemotherapy in ovarian cancer patients (40).

With the extended set of seed genes, a drug search using harmonic centrality was performed. The GGI dataset BioGRID as well as the DGI dataset DrugBank were used. To receive more explorative results, indirect and non-approved drugs were included while nutraceutical drugs were excluded to find more case-relevant drugs. In addition, the drugs were reduced to only CTRPv2 drugs. The result size was limited to 20 drugs with the highest scores (see Figure S5).

Analyzing the drug result, we notice that none of the seed genes has a direct drug interaction in the DrugBank dataset. However, CADDIE identified drugs with an impact on the given disease module by detecting mechanisms of co-regulation. One of the highest scored drugs is alvocidib, a cyclin-dependent kinase inhibitor (41) and second-order neighbor to three of the seed genes. Alvocidib is an investigational drug that has successfully passed phase I clinical trials (42). Among its targets is CDK1, a member of the cyclin-dependent kinases, which is predicted by CADDIE to affect MYC (see Figure S5), similar to the predictions by Zeng et al. (38). Staurosporine is one more investigational drug represented in the top-scored drugs (see Table S1). It has been shown to improve outcomes of chemotherapy in ovarian cancer in vitro (43) as well as in mouse models (44), although its mechanisms remain unclear. CADDIE predicts its effect on the seed genes MYC, CASP3, and BRCA1 by targeting the genes GSK3B, CDK2, and LCK, respectively. Out of the remaining best scored drugs, tamoxifen (45), paclitaxel (46), and docetaxel (47) are known for treatment of ovarian cancer. Bosutinib is not applied in ovarian cancer but received a high score in CADDIE, making it a putative drug for further research.

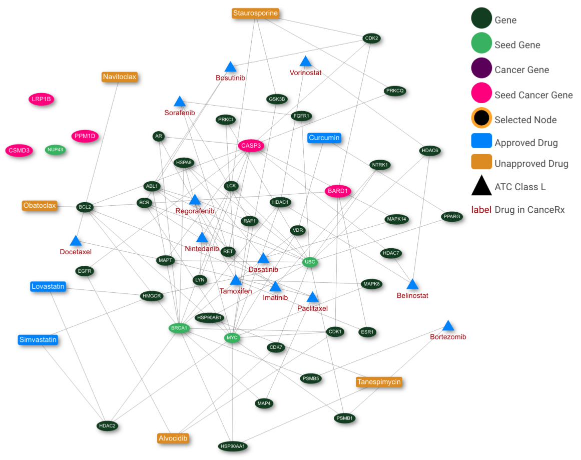

Figure S5: Drug network computed with harmonic centrality based on COSMIC cancer driver genes and DrugBank drug-gene interactions.

Table S1: Drug search results for ovarian cancer

Name	Approved	ATC L	CanceRx	Score	Degree
Alvocidib	no	no	no	1	11
Staurosporine	no	no	no	1	13
Tamoxifen	yes	yes	yes	1	17
Bosutinib	yes	yes	yes	1	10
Paclitaxel	yes	yes	yes	1	6
Docetaxel	yes	yes	yes	1	6
Dasatinib	yes	yes	yes	0.96	24
Regorafenib	yes	yes	yes	0.96	18
Belinostat	yes	yes	yes	0.96	11
Obatoclax	no	no	no	0.92	1
Navitoclax	no	no	no	0.92	3
Imatinib	yes	yes	yes	0.91	2
Simvastatin	yes	no	yes	0.91	3
Bortezomib	yes	yes	yes	0.91	2
Curcumin	yes	no	no	0.91	5
Vorinostat	yes	yes	yes	0.91	5
Nintedanib	yes	yes	yes	0.91	12
Lovastatin	yes	no	yes	0.91	3
Tanespimycin	no	no	no	0.91	2
Sorafenib	yes	yes	yes	0.91	10

Listed are the top 20 drug results for ovarian cancer. Each row contains the drug name, approval by FDA, EMA or HC, whether it is listed as antineoplastic or immunomodulating agent (ATC class L) by the WHO, whether it is contained in CanceRx, the normalized score and the node degree in the drug-gene-interactome (DrugBank).

Low node degrees increase the relevance of the found node since they are less likely to be reported by chance. Obatoclax is found amongst the highest scoring drugs with only one interaction to BCL2, which shows interactions with three of the original seed genes. Furthermore, it is known to participate in apoptosis in epithelial ovarian cancer cells (48). Hence, BCL2 represents a potentially valuable target. In addition to obatoclax, CADDIE proposes navitoclax as a drug to target BCL2, an investigational drug to reduce BCL-induced resistance to chemotherapy in ovarian cancer (49).

References

1. Peixoto, T. P. (2014) The graph-tool python library. *figshare,*.

2. Repana, D., Nulsen, J., Dressler, L., Bortolomeazzi, M., Venkata, S. K., Tourna, A., Yakovleva, A., Palmieri, T., and Ciccarelli, F. D. (1, 2019) The Network of Cancer Genes (NCG): A comprehensive catalogue of known and candidate cancer genes from cancer sequencing screens 06 Biological Sciences 0604 Genetics 11 Medical and Health Sciences 1112 Oncology and Carcinogenesis 06 Biological Sciences 0601 Biochemistry and Cell Biology. *Genome Biology,* **20**, 1–12.

3. Tate, J. G., Bamford, S., Jubb, H. C., Sondka, Z., Beare, D. M., Bindal, N., Boutselakis, H., Cole, C. G., Creatore, C., Dawson, E., Fish, P., Harsha, B., Hathaway, C., Jupe, S. C., Kok, C. Y., Noble, K., Ponting, L., Ramshaw, C. C., Rye, C. E., Speedy, H. E., Stefancsik, R., Thompson, S. L., Wang, S., Ward, S., Campbell, P. J., and Forbes, S. A. (1, 2019) COSMIC: the Catalogue Of Somatic Mutations In Cancer. *Nucleic Acids Research,* **47**, D941–D947.

4. Martínez-Jiménez, F., Muiños, F., Sentís, I., Deu-Pons, J., Reyes-Salazar, I., Arnedo-Pac, C., Mularoni, L., Pich, O., Bonet, J., Kranas, H., Gonzalez-Perez, A., and Lopez-Bigas, N. (8, 2020) A compendium of mutational cancer driver genes. *Nature Reviews Cancer 2020 20:10,* **20**, 555–572 [PubMed:32778778] [doi:10.1038/s41568-020-0290-x].

5. Dietlein, F., Weghorn, D., Taylor-Weiner, A., Richters, A., Reardon, B., Liu, D., Lander, E. S., Allen, E. M. V., and Sunyaev, S. R. (2, 2020) Identification of cancer driver genes based on nucleotide context. *Nature Genetics 2020 52:2,* **52**, 208–218 [PubMed:32015527] [PubMed Central:PMC7031046] [doi:10.1038/s41588-019-0572-y].

6. Oughtred, R., Rust, J., Chang, C., Breitkreutz, B. J., Stark, C., Willems, A., Boucher, L., Leung, G., Kolas, N., Zhang, F., Dolma, S., Coulombe-Huntington, J., Chatr-aryamontri, A., Dolinski, K., and Tyers, M. (1, 2021) The BioGRID database: A comprehensive biomedical resource of curated protein, genetic, and chemical interactions. *Protein science : a publication of the Protein Society,* **30**, 187–200 [PubMed:33070389] [PubMed Central:PMC7737760] [doi:10.1002/pro.3978].

7. Szklarczyk, D., Gable, A. L., Lyon, D., Junge, A., Wyder, S., Huerta-Cepas, J., Simonovic, M., Doncheva, N. T., Morris, J. H., Bork, P., Jensen, L. J., and Mering, C. V. (1, 2019) STRING v11: protein-protein association networks with increased coverage, supporting functional discovery in genome-wide experimental datasets. *Nucleic acids research,* **47**, D607–D613 [PubMed:30476243] [PubMed Central:PMC6323986] [doi:10.1093/nar/gky1131].

8. Alonso-López, D., Campos-Laborie, F. J., Gutiérrez, M. A., Lambourne, L., Calderwood, M. A., Vidal, M., and Rivas, J. D. L. (1, 2019) APID database: redefining protein–protein interaction experimental evidences and binary interactomes. *Database: The Journal of Biological Databases and Curation,* **2019**, 5 [PubMed:30715274] [PubMed Central:PMC6354026] [doi:10.1093/database/baz005].

9. Kotlyar, M., Pastrello, C., Ahmed, Z., Chee, J., Varyova, Z., and Jurisica, I. (1, 2022) IID 2021: towards context-specific protein interaction analyses by increased coverage, enhanced annotation and enrichment analysis. *Nucleic Acids Research,* **50**, D640–D647 [PubMed:34755877] [PubMed Central:PMC8728267] [doi:10.1093/nar/gkab1034].

10. Bovolenta, L. A., Acencio, M. L., and Lemke, N. (8, 2012) HTRIdb: an open-access database for experimentally verified human transcriptional regulation interactions. *BMC Genomics,* **13**, 1–10 [PubMed:22900683] [PubMed Central:PMC3472291] [doi:10.1186/1471-2164-13-405].

11. Jassal, B., Matthews, L., Viteri, G., Gong, C., Lorente, P., Fabregat, A., Sidiropoulos, K., Cook, J., Gillespie, M., Haw, R., Loney, F., May, B., Milacic, M., Rothfels, K., Sevilla, C., Shamovsky, V., Shorser, S., Varusai, T., Weiser, J., Wu, G., Stein, L., Hermjakob, H., and D'Eustachio, P. (1, 2020) The reactome pathway knowledgebase. *Nucleic acids research,* **48**, D498–D503.

12. Wishart, D. S., Feunang, Y. D., Guo, A. C., Lo, E. J., Marcu, A., Grant, J. R., Sajed, T., Johnson, D., Li, C., Sayeeda, Z., Assempour, N., Iynkkaran, I., Liu, Y., MacIejewski, A., Gale, N., Wilson, A., Chin, L., Cummings, R., Le, D., Pon, A., Knox, C., and Wilson, M. (1, 2018) DrugBank 5.0: a major update to the DrugBank database for 2018. *Nucleic acids research,* **46**, D1074–D1082 [PubMed:29126136] [PubMed Central:PMC5753335] [doi:10.1093/nar/gkx1037].

13. Gaulton, A., Hersey, A., Nowotka, M. L., Bento, A. P., Chambers, J., Mendez, D., Mutowo, P., Atkinson, F., Bellis, L. J., Cibrian-Uhalte, E., Davies, M., Dedman, N., Karlsson, A., Magarinos, M. P., Overington, J. P., Papadatos, G., Smit, I., and Leach, A. R. (1, 2017) The ChEMBL database in 2017. *Nucleic acids research,* **45**, D945–D954 [PubMed:27899562] [PubMed Central:PMC5210557] [doi:10.1093/nar/gkw1074].

14. Cotto, K. C., Wagner, A. H., Feng, Y. Y., Kiwala, S., Coffman, A. C., Spies, G., Wollam, A., Spies, N. C., Griffith, O. L., and Griffith, M. (1, 2018) DGIdb 3.0: a redesign and expansion of the drug–gene interaction database. *Nucleic Acids Research,* **46**, D1068–D1073 [PubMed:29156001] [PubMed Central:PMC5888642] [doi:10.1093/nar/gkx1143].

15. Sadegh, S., Skelton, J., Anastasi, E., Bernett, J., Blumenthal, D. B., Galindez, G., Salgado-Albarrán, M., Lazareva, O., Flanagan, K., Cockell, S., Nogales, C., Casas, A. I., Schmidt, H. H., Baumbach, J., Wipat, A., and Kacprowski, T. (11, 2021) Network medicine for disease module identification and drug repurposing with the NeDRex platform. *Nature Communications 2021 12:1,* **12**, 1–12 [PubMed:34824199] [PubMed Central:PMC8617287] [doi:10.1038/s41467-021-27138-2].

16. Grossman, R. L., Heath, A. P., Ferretti, V., Varmus, H. E., Lowy, D. R., Kibbe, W. A., and Staudt, L. M. (9, 2016) Toward a Shared Vision for Cancer Genomic Data. *The New England journal of medicine,* **375**, 1109–1112 [PubMed:27653561] [PubMed Central:PMC6309165] [doi:10.1056/NEJMp1607591].

17. Ellrott, K., Bailey, M. H., Saksena, G., Covington, K. R., Kandoth, C., Stewart, C., Hess, J., Ma, S., Chiotti, K. E., McLellan, M. D., Sofia, H. J., Hutter, C. M., Getz, G., Wheeler, D. A., Ding, L., Caesar-Johnson, S. J., Demchok, J. A., Felau, I., Kasapi, M., Ferguson, M. L., Tarnuzzer, R., Wang, Z., Yang, L., Zenklusen, J. C., Zhang, J. J., Chudamani, S., Liu, J., Lolla, L., Naresh, R., Pihl, T., Sun, Q., Wan, Y., Wu, Y., Cho, J., DeFreitas, T., Frazer, S., Gehlenborg, N., Heiman, D. I., Kim, J., Lawrence, M. S., Lin, P., Meier, S., Noble, M. S., Voet, D., Zhang, H., Bernard, B., Chambwe, N., Dhankani, V., Knijnenburg, T., Kramer, R., Leinonen, K., Liu, Y., Miller, M., Reynolds, S., Shmulevich, I., Thorsson, V., Zhang, W., Akbani, R., Broom, B. M., Hegde, A. M., Ju, Z., Kanchi, R. S., Korkut, A., Li, J., Liang, H., Ling, S., Liu, W., Lu, Y., Mills, G. B., Ng, K. S., Rao, A., Ryan, M., Wang, J., Weinstein, J. N., Zhang, J., Abeshouse, A., Armenia, J., Chakravarty, D., Chatila, W. K., de Bruijn, I., Gao, J., Gross, B. E., Heins, Z. J., Kundra, R., La, K., Ladanyi, M., Luna, A., Nissan, M. G., Ochoa, A., Phillips, S. M., Reznik, E., Sanchez-Vega, F., Sander, C., Schultz, N., Sheridan, R., Sumer, S. O., Sun, Y., Taylor, B. S., Wang, J., Zhang, H., Anur, P., Peto, M., Spellman, P., Benz, C., Stuart, J. M., Wong, C. K., Yau, C., Hayes, D. N., Parker, Ally, A., Balasundaram, M., Bowlby, R., Brooks, D., Carlsen, R., Chuah, E., Dhalla, N., Holt, R., Jones, S. J., Kasaian, K., Wilkerson, M. D., Ma, Y., Marra, M. A., Mayo, M., Moore, R. A., Mungall, A. J., Mungall, K., Robertson, A. G., Sadeghi, S., Schein, J. E., Sipahimalani, P., Tam, A., Thiessen, N., Tse, K., Wong, T., Berger, A. C., Beroukhim, R., Cherniack, A. D., Cibulskis, C., Gabriel, S. B., Gao, G. F., Ha, G., Meyerson, M., Schumacher, S. E., Shih, J., Kucherlapati, M. H., Kucherlapati, R. S., Baylin, S., Cope, L., Danilova, L., Bootwalla, M. S., Lai, P. H., Maglinte, D. T., Berg, D. J. V. D., Weisenberger, D. J., Auman, J. T., Balu, S., Bodenheimer, T., Fan, C., Hoadley, K. A., Hoyle, A. P., Jefferys, S. R., Jones, C. D., Meng, S., Mieczkowski, P. A., Mose, L. E., Perou, A. H., Perou, C. M., Roach, J., Shi, Y., Simons, J. V., Skelly, T., Soloway, M. G., Tan, D., Veluvolu, U., Fan, H., Hinoue, T., Laird, P. W., Shen, H., Zhou, W., Bellair, M., Chang, K., Creighton, C. J., Dinh, H., Doddapaneni, H. V., Donehower, L. A., Drummond, J., Gibbs, R. A., Glenn, R., Hale, W., Han, Y., Hu, J., Korchina, V., Lee, S., Lewis, L., Li, W., Liu, X., Morgan, M., Morton, D., Muzny, D., Santibanez, J., Sheth, M., Shinbrot, E., Wang, L., Wang, M., Xi, L., Zhao, F., Appelbaum, E. L., Cordes, M. G., Fronick, C. C., Fulton, L. A., Fulton, R. S., Mardis, E. R., Miller, C. A., Schmidt, H. K., Wilson, R. K., Crain, D., Curley, E., Gardner, J., Lau, K., Mallery, D., Morris, S., Paulauskis, J., Penny, R., Shelton, C., Shelton, T., Sherman, M., Thompson, E., Yena, P., Bowen, J., Gastier-Foster, J. M., Gerken, M., Leraas, K. M., Lichtenberg, T. M., Ramirez, N. C., Wise, L., Zmuda, E., Corcoran, N., Costello, T., Hovens, C., Carvalho, A. L., de Carvalho, A. C., Fregnani, J. H., Longatto-Filho, A., Reis, R. M., Scapulatempo-Neto, C., Silveira, H. C., Vidal, D. O., Burnette, A., Eschbacher, J., Hermes, B., Noss, A., Singh, R., Anderson, M. L., Castro, P. D., Ittmann, M., Huntsman, D., Kohl, B., Le, X., Thorp, R., Andry, C., Duffy, E. R., Lyadov, V., Paklina, O., Setdikova, G., Shabunin, A., Tavobilov, M., McPherson, C., Warnick, R., Berkowitz, R., Cramer, D., Feltmate, C., Horowitz, N., Kibel, A., Muto, M., Raut, C. P., Malykh, A., Barnholtz-Sloan, J. S., Barrett, W., Devine, K., Fulop, J., Ostrom, Q. T., Shimmel, K., Wolinsky, Y., Sloan, A. E., Rose, A. D., Giuliante, F., Goodman, M., Karlan, B. Y., Hagedorn, C. H., Eckman, J., Harr, J., Myers, J., Tucker, K., Zach, L. A., Deyarmin, B., Hu, H., Kvecher, L., Larson, C., Mural, R. J., Somiari, S., Vicha, A., Zelinka, T., Bennett, J., Iacocca, M., Rabeno, B., Swanson, P., Latour, M., Lacombe, L., Têtu, B., Bergeron, A., McGraw, M., Staugaitis, S. M., Chabot, J., Hibshoosh,

H., Sepulveda, A., Su, T., Wang, T., Potapova, O., Voronina, O., Desjardins, L., Mariani, O., Roman-Roman, S., Sastre, X., Stern, M. H., Cheng, F., Signoretti, S., Berchuck, A., Bigner, D., Lipp, E., Marks, J., McCall, S., McLendon, R., Secord, A., Sharp, A., Behera, M., Brat, D. J., Chen, A., Delman, K., Force, S., Khuri, F., Magliocca, K., Maithel, S., Olson, J. J., Owonikoko, T., Pickens, A., Ramalingam, S., Shin, D. M., Sica, G., Meir, E. G. V., Zhang, H., Eijckenboom, W., Gillis, A., Korpershoek, E., Looijenga, L., Oosterhuis, W., Stoop, H., van Kessel, K. E., Zwarthoff, E. C., Calatozzolo, C., Cuppini, L., Cuzzubbo, S., DiMeco, F., Finocchiaro, G., Mattei, L., Perin, A., Pollo, B., Chen, C., Houck, J., Lohavanichbutr, P., Hartmann, A., Stoehr, C., Stoehr, R., Taubert, H., Wach, S., Wullich, B., Kycler, W., Murawa, D., Wiznerowicz, M., Chung, K., Edenfield, W. J., Martin, J., Baudin, E., Bubley, G., Bueno, R., Rienzo, A. D., Richards, W. G., Kalkanis, S., Mikkelsen, T., Noushmehr, H., Scarpace, L., Girard, N., Aymerich, M., Campo, E., Giné, E., Guillermo, A. L., Bang, N. V., Hanh, P. T., Phu, B. D., Tang, Y., Colman, H., Evason, K., Dottino, P. R., Martignetti, J. A., Gabra, H., Juhl, H., Akeredolu, T., Stepa, S., Hoon, D., Ahn, K., Kang, K. J., Beuschlein, F., Breggia, A., Birrer, M., Bell, D., Borad, M., Bryce, A. H., Castle, E., Chandan, V., Cheville, J., Copland, J. A., Farnell, M., Flotte, T., Giama, N., Ho, T., Kendrick, M., Kocher, J. P., Kopp, K., Moser, C., Nagorney, D., O'Brien, D., O'Neill, B. P., Patel, T., Petersen, G., Que, F., Rivera, M., Roberts, L., Smallridge, R., Smyrk, T., Stanton, M., Thompson, R. H., Torbenson, M., Yang, J. D., Zhang, L., Brimo, F., Ajani, J. A., Gonzalez, A. M. A., Behrens, C., Bondaruk, J., Broaddus, R., Czerniak, B., Esmaeli, B., Fujimoto, J., Gershenwald, J., Guo, C., Lazar, A. J., Logothetis, C., Meric-Bernstam, F., Moran, C., Ramondetta, L., Rice, D., Sood, A., Tamboli, P., Thompson, T., Troncoso, P., Tsao, A., Wistuba, I., Carter, C., Haydu, L., Hersey, P., Jakrot, V., Kakavand, H., Kefford, R., Lee, K., Long, G., Mann, G., Quinn, M., Saw, R., Scolyer, R., Shannon, K., Spillane, A., Stretch, J., Synott, M., Thompson, J., Wilmott, J., Al-Ahmadie, H., Chan, T. A., Ghossein, R., Gopalan, A., Levine, D. A., Reuter, V., Singer, S., Singh, B., Tien, N. V., Broudy, T., Mirsaidi, C., Nair, P., Drwiega, P., Miller, J., Smith, J., Zaren, H., Park, J. W., Hung, N. P., Kebebew, E., Linehan, W. M., Metwalli, A. R., Pacak, K., Pinto, P. A., Schiffman, M., Schmidt, L. S., Vocke, C. D., Wentzensen, N., Worrell, R., Yang, H., Moncrieff, M., Goparaju, C., Melamed, J., Pass, H., Botnariuc, N., Caraman, I., Cernat, M., Chemencedji, I., Clipca, A., Doruc, S., Gorincioi, G., Mura, S., Pirtac, M., Stancul, I., Tcaciuc, D., Albert, M., Alexopoulou, I., Arnaout, A., Bartlett, J., Engel, J., Gilbert, S., Parfitt, J., Sekhon, H., Thomas, G., Rassl, D. M., Rintoul, R. C., Bifulco, C., Tamakawa, R., Urba, W., Hayward, N., Timmers, H., Antenucci, A., Facciolo, F., Grazi, G., Marino, M., Merola, R., de Krijger, R., Gimenez-Roqueplo, A. P., Piché, A., Chevalier, S., McKercher, G., Birsoy, K., Barnett, G., Brewer, C., Farver, C., Naska, T., Pennell, N. A., Raymond, D., Schilero, C., Smolenski, K., Williams, F., Morrison, C., Borgia, J. A., Liptay, M. J., Pool, M., Seder, C. W., Junker, K., Omberg, L., Dinkin, M., Manikhas, G., Alvaro, D., Bragazzi, M. C., Cardinale, V., Carpino, G., Gaudio, E., Chesla, D., Cottingham, S., Dubina, M., Moiseenko, F., Dhanasekaran, R., Becker, K. F., Janssen, K. P., Slotta-Huspenina, J., Abdel-Rahman, M. H., Aziz, D., Bell, S., Cebulla, C. M., Davis, A., Duell, R., Elder, J. B., Hilty, J., Kumar, B., Lang, J., Lehman, N. L., Mandt, R., Nguyen, P., Pilarski, R., Rai, K., Schoenfield, L., Senecal, K., Wakely, P., Hansen, P., Lechan, R., Powers, J., Tischler, A., Grizzle, W. E., Sexton, K. C., Kastl, A., Henderson, J., Porten, S., Waldmann, J., Fassnacht, M., Asa, S. L., Schadendorf, D., Couce, M., Graefen, M., Huland, H., Sauter, G., Schlomm, T., Simon, R., Tennstedt, P., Olabode, O., Nelson, M., Bathe, O., Carroll, P. R., Chan, J. M., Disaia, P., Glenn, P., Kelley, R. K., Landen, C. N., Phillips, J., Prados, M., Simko, J., Smith-McCune, K., VandenBerg, S., Roggin, K., Fehrenbach, A., Kendler, A., Sifri, S., Steele, R., Jimeno, A., Carey, F., Forgie, I., Mannelli, M., Carney, M., Hernandez, B., Campos, B., Herold-Mende, C., Jungk, C., Unterberg, A., von Deimling, A., Bossler, A., Galbraith, J., Jacobus, L., Knudson, M., Knutson, T., Ma, D., Milhem, M., Sigmund, R., Godwin, A. K., Madan, R., Rosenthal, H. G., Adebamowo, C., Adebamowo, S. N., Boussioutas, A., Beer, D., Giordano, T., Mes-Masson, A. M., Saad, F., Bocklage, T., Landrum, L., Mannel, R., Moore, K., Moxley, K., Postier, R., Walker, J., Zuna, R., Feldman, M., Valdivieso, F., Dhir, R., Luketich, J., Pinero, E. M. M., Quintero-Aguilo, M., Carlotti, C. G., Santos, J. S. D., Kemp, R., Sankarankuty, A., Tirapelli, D., Catto, J., Agnew, K., Swisher, E., Creaney, J., Robinson, B., Shelley, C. S., Godwin, E. M., Kendall, S., Shipman, C., Bradford, C., Carey, T., Haddad, A., Moyer, J., Peterson, L., Prince, M., Rozek, L., Wolf, G., Bowman, R., Fong, K. M., Yang, I., Korst, R., Rathmell, W. K., Fantacone-Campbell, J. L., Hooke, J. A., Kovatich, A. J., Shriver, C. D., DiPersio, J., Drake, B., Govindan, R., Heath, S., Ley, T., Tine, B. V., Westervelt, P., Rubin, M. A., Lee, J. I., Aredes, N. D., and Mariamidze, A. (3, 2018) Scalable Open Science Approach for Mutation Calling of Tumor Exomes Using Multiple Genomic Pipelines. *Cell Systems,* **6**, 271–281.e7 [PubMed:29596782] [PubMed Central:PMC6075717] [doi:10.1016/j.cels.2018.03.002].

18. Lonsdale, J., Thomas, J., Salvatore, M., Phillips, R., Lo, E., Shad, S., Hasz, R., Walters, G., Garcia, F., Young, N., Foster, B., Moser, M., Karasik, E., Gillard, B., Ramsey, K., Sullivan, S., Bridge, J., Magazine, H., Syron, J., Fleming, J., Siminoff, L., Traino, H., Mosavel, M., Barker, L., Jewell, S., Rohrer, D., Maxim, D., Filkins, D.,

Harbach, P., Cortadillo, E., Berghuis, B., Turner, L., Hudson, E., Feenstra, K., Sobin, L., Robb, J., Branton, P., Korzeniewski, G., Shive, C., Tabor, D., Qi, L., Groch, K., Nampally, S., Buia, S., Zimmerman, A., Smith, A., Burges, R., Robinson, K., Valentino, K., Bradbury, D., Cosentino, M., Diaz-Mayoral, N., Kennedy, M., Engel, T., Williams, P., Erickson, K., Ardlie, K., Winckler, W., Getz, G., DeLuca, D., MacArthur, D., Kellis, M., Thomson, A., Young, T., Gelfand, E., Donovan, M., Meng, Y., Grant, G., Mash, D., Marcus, Y., Basile, M., Liu, J., Zhu, J., Tu, Z., Cox, N. J., Nicolae, D. L., Gamazon, E. R., Im, H. K., Konkashbaev, A., Pritchard, J., Stevens, M., Flutre, T., Wen, X., Dermitzakis, E. T., Lappalainen, T., Guigo, R., Monlong, J., Sammeth, M., Koller, D., Battle, A., Mostafavi, S., McCarthy, M., Rivas, M., Maller, J., Rusyn, I., Nobel, A., Wright, F., Shabalin, A., Feolo, M., Sharopova, N., Sturcke, A., Paschal, J., Anderson, J. M., Wilder, E. L., Derr, L. K., Green, E. D., Struewing, J. P., Temple, G., Volpi, S., Boyer, J. T., Thomson, E. J., Guyer, M. S., Ng, C., Abdallah, A., Colantuoni, D., Insel, T. R., Koester, S. E., Little, A. R., Bender, P. K., Lehner, T., Yao, Y., Compton, C. C., Vaught, J. B., Sawyer, S., Lockhart, N. C., Demchok, J., and Moore, H. F. (6, 2013) The Genotype-Tissue Expression (GTEx) project. *Nature genetics,* **45**, 580.

19. Tokheim, C. J., Papadopoulos, N., Kinzler, K. W., Vogelstein, B., and Karchin, R. (12, 2016) Evaluating the evaluation of cancer driver genes. *Proceedings of the National Academy of Sciences of the United States of America,* **113**, 14330–14335 [PubMed:27911828] [PubMed Central:PMC5167163] [doi:10.1073/pnas.1616440113].

20. Cordell, H. J. (6, 2009) Detecting gene-gene interactions that underlie human diseases. *Nature reviews. Genetics,* **10**, 392–404 [PubMed:19434077] [PubMed Central:PMC2872761] [doi:10.1038/nrg2579].

21. Guney, E., Menche, J., Vidal, M., and Barábasi, A. L. (2, 2016) Network-based in silico drug efficacy screening. *Nature Communications 2016 7:1,* **7**, 1–13 [PubMed:26831545] [PubMed Central:PMC4740350] [doi:10.1038/ncomms10331].

22. [PDF] Closeness Centrality Extended to Unconnected Graphs: the Harmonic Centrality Index — Semantic Scholar.

23. Zhang, H., Fiszman, M., Shin, D., Miller, C. M., Rosemblat, G., and Rindflesch, T. C. (10, 2011) Degree centrality for semantic abstraction summarization of therapeutic studies. *Journal of biomedical informatics,* **44**, 830–838 [PubMed:21575741] [PubMed Central:PMC3170688] [doi:10.1016/j.jbi.2011.05.001].

24. Tang, X., Wang, J., Zhong, J., and Pan, Y. (2014) Predicting essential proteins basedon weighted degree centrality. *IEEE/ACM Transactions on Computational Biology and Bioinformatics,* **11**, 407–418.

25. Okamoto, K., Chen, W., and Li, X. Y. (2008) Ranking of closeness centrality for large-scale social networks. *Lecture Notes in Computer Science (including subseries Lecture Notes in Artificial Intelligence and Lecture Notes in Bioinformatics),* **5059 LNCS**, 186–195.

26. Marchiori, M. and Latora, V. (8, 2000) Harmony in the Small-World. *Physica A: Statistical Mechanics and its Applications,* **285**, 539–546.

27. Koschützki, D. and Schreiber, F. (2008) Centrality analysis methods for biological networks and their application to gene regulatory networks. *Gene Regulation and Systems Biology,* **2008**, 193–201 [PubMed:19787083] [PubMed Central:PMC2733090] [doi:10.4137/grsb.s702].

28. Jalili, M., Salehzadeh-Yazdi, A., Gupta, S., Wolkenhauer, O., Yaghmaie, M., Resendis-Antonio, O., and Alimoghaddam, K. (8, 2016) Evolution of Centrality Measurements for the Detection of Essential Proteins in Biological Networks. *Frontiers in physiology,* **7** [PubMed:27616995] [PubMed Central:PMC4999434] [doi:10.3389/fphys.2016.00375].

29. Newman, M. E. (1, 2005) A measure of betweenness centrality based on random walks. *Social Networks,* **27**, 39–54.

30. Junker, B. H., Koschützki, D., and Schreiber, F. (4, 2006) Exploration of biological network centralities with CentiBiN. *BMC Bioinformatics,* **7** [PubMed:16630347] [PubMed Central:PMC1524990] [doi:10.1186/1471-2105-7-219].

31. Girvan, M. and Newman, M. E. (6, 2002) Community structure in social and biological networks. *Proceedings of the National Academy of Sciences of the United States of America,* **99**, 7821–7826 [PubMed:12060727] [PubMed Central:PMC122977] [doi:10.1073/pnas.122653799].

32. Kacprowski, T., Doncheva, N. T., and Albrecht, M. (6, 2013) NetworkPrioritizer: a versatile tool for network-based prioritization of candidate disease genes or other molecules. *Bioinformatics,* **29**, 1471–1473.

33. Alexeyenko, A., Lee, W., Pernemalm, M., Guegan, J., Dessen, P., Lazar, V., Lehtiö, J., and Pawitan, Y. (9, 2012) Network enrichment analysis: extension of gene-set enrichment analysis to gene networks. *BMC Bioinformatics,* **13**, 1–11 [PubMed:22966941] [PubMed Central:PMC3505158] [doi:10.1186/1471-2105-13-226].

34. Alcaraz, N., List, M., Dissing-Hansen, M., Rehmsmeier, M., Tan, Q., Mollenhauer, J., Ditzel, H. J., and Baumbach, J. (2016) Robust de novo pathway enrichment with KeyPathwayMiner 5 [version 1; referees: 2 approved]. *F1000Research,* **5**.

35. Graham, R. L. and Hell, P. (1985) On the History of the Minimum Spanning Tree Problem. *Annals of the History of Computing,* **7**, 43–57.

36. Ahmed, R., Angelini, P., Sahneh, F. D., Efrat, A., Glickenstein, D., Gronemann, M., Heinsohn, N., Kobourov, S. G., Spence, R., Watkins, J., and Wolff, A. (12, 2019) Multi-level Steiner Trees. *Journal of Experimental Algorithmics (JEA),* **24**.

37. Petrucelli, N., Daly, M. B., and Pal, T. (2, 2022) BRCA1- and BRCA2-Associated Hereditary Breast and Ovarian Cancer. *GeneReviews®,*.

38. Zeng, M., Kwiatkowski, N. P., Zhang, T., Nabet, B., Xu, M., Liang, Y., Quan, C., Wang, J., Hao, M., Palakurthi, S., Zhou, S., Zeng, Q., Kirschmeier, P. T., Meghani, K., Leggett, A. L., Qi, J., Shapiro, G. I., Liu, J. F., Matulonis, U. A., Lin, C. Y., Konstantinopoulos, P. A., and Gray, N. S. (11, 2018) Targeting MYC dependency in ovarian cancer through inhibition of CDK7 and CDK12/13. *eLife,* **7** [PubMed:30422115] [PubMed Central:PMC6251623] [doi:10.7554/eLife.39030].

39. Kedves, A. T., Gleim, S., Liang, X., Bonal, D. M., Sigoillot, F., Harbinski, F., Sanghavi, S., Benander, C., George, E., Gokhale, P. C., Nguyen, Q. D., Kirschmeier, P. T., Distel, R. J., Jenkins, J., Goldberg, M. S., and Forrester, W. C. (12, 2017) Recurrent ubiquitin B silencing in gynecological cancers establishes dependence on ubiquitin C. *The Journal of clinical investigation,* **127**, 4554–4568 [PubMed:29130934] [PubMed Central:PMC5707153] [doi:10.1172/JCI92914].

40. Alanee, S., Delfino, K., Wilber, A., Robinson, K., Brard, L., and Semaan, A. (2017) Single Nucleotide Variant in Nucleoporin 107 may be Predictive of Sensitivity to Chemotherapy in Patient with Ovarian Cancer. *Pharmacogenetics and genomics,* **27**, 264.

41. Baumann, K. H., Kim, H., Rinke, J., Plaum, T., Wagner, U., and Reinartz, S. (2013) Effects of alvocidib and carboplatin on ovarian cancer cells in vitro. *Experimental oncology,* pp. 168–173 [PubMed:24084453].

42. Holkova, B., Perkins, E. B., Ramakrishnan, V., Tombes, M. B., Shrader, E., Talreja, N., Wellons, M. D., Hogan, K. T., Roodman, G. D., Coppola, D., Kang, L., Dawson, J., Stuart, R. K., Peer, C., Figg, W. D., Kolla, S., Doyle, A., Wright, J., Sullivan, D. M., Roberts, J. D., and Grant, S. (5, 2011) Phase I trial of bortezomib (PS-341; NSC 681239) and alvocidib (flavopiridol; NSC 649890) in patients with recurrent or refractory B-cell neoplasms. *Clinical Cancer Research,* **17**, 3388–3397.

43. Alsamman, K. and El-Masry, O. S. (10, 2018) Staurosporine alleviates cisplatin chemoresistance in human cancer cell models by suppressing the induction of SQSTM1/p62. *Oncology Reports,* **40**, 2157–2162 [PubMed:30106434] [doi:10.3892/or.2018.6615].

44. Huang, Z., Kondoh, E., Visco, Z. R., Baba, T., Matsumura, N., Dolan, E., Whitaker, R. S., Konishi, I., Fujii, S., Berchuck, A., and Murphy, S. K. (1, 2021) Targeting Dormant Ovarian Cancer Cells In Vitro and in an In Vivo Mouse Model of Platinum Resistance. *Molecular cancer therapeutics,* **20**, 85–95 [PubMed:33037137] [doi:10.1158/1535-7163.MCT-20-0119].

45. Karagol, H., Saip, P., Uygun, K., Caloglu, M., Eralp, Y., Tas, F., Aydiner, A., and Topuz, E. (6, 2007) The efficacy of tamoxifen in patients with advanced epithelial ovarian cancer. *Medical Oncology 2007 24:1,* **24**, 39–43 [PubMed:17673810] [doi:10.1007/BF02685901].

46. Kampan, N. C., Madondo, M. T., McNally, O. M., Quinn, M., and Plebanski, M. (2015) Paclitaxel and its evolving role in the management of ovarian cancer. *BioMed Research International,* **2015** [PubMed:26137480] [PubMed Central:PMC4475536] [doi:10.1155/2015/413076].

47. Katsumata, N. (12, 2003) Docetaxel: an alternative taxane in ovarian cancer. *British Journal of Cancer 2003 89:3,* **89**, S9–S15 [PubMed:14661041] [PubMed Central:PMC2750619] [doi:10.1038/sj.bjc.6601495].

48. Yasmeen, A., Beauchamp, M. C., Piura, E., Segal, E., Pollak, M., and Gotlieb, W. H. (6, 2011) Induction of apoptosis by metformin in epithelial ovarian cancer: Involvement of the Bcl-2 family proteins. *Gynecologic Oncology,* **121**, 492–498 [PubMed:21388661] [doi:10.1016/j.ygyno.2011.02.021].

49. Wong, M., Tan, N., Zha, J., Peale, F. V., Yue, P., Fairbrother, W. J., and Belmont, L. D. (2012) Navitoclax (ABT-263) reduces Bcl-x(L)-mediated chemoresistance in ovarian cancer models. *Molecular cancer therapeutics,* **11**, 1026–1035 [PubMed:22302098] [doi:10.1158/1535-7163.MCT-11-0693].
